# Wafer‐Scale Room‐Temperature Processing of Lead‐Free Perovskites for Optoelectronic Applications

**DOI:** 10.1002/advs.202517469

**Published:** 2026-02-09

**Authors:** Rosanna Mastria, Hoi Tung Lam, Ioannis Leontis, Sara Bonomi, Mohammed Ali Saleh Alshehri, Aurora Rizzo, Pietro Galinetto, Maddalena Patrini, Ned Taylor, Steven P. Hepplestone, Monica F. Craciun, Lorenzo Malavasi, Saverio Russo

**Affiliations:** ^1^ Centre for Graphene Science, Department of Physics and Astronomy University of Exeter Exeter UK; ^2^ Department of Chemistry and INSTM University of Pavia Pavia Italy; ^3^ CNR NANOTEC Institute of Nanotechnology Lecce Italy; ^4^ Department of Physics University of Pavia Pavia Italy; ^5^ Centre for Graphene Science Department of Engineering University of Exeter Exeter UK

**Keywords:** lead free perovskites, photodetectors, wafer scale manufacturing

## Abstract

Scalable and sustainable synthesis methods for high‐performance optoelectronic materials are essential to the advancement of next‐generation optoelectronic and photonic technologies. Metal halide perovskites have rapidly gained prominence as versatile semiconductors across applications ranging from photovoltaics to lighting and sensing. However, their commercial deployment is hindered by the pervasive use of toxic lead‐based compositions and the lack of environmentally friendly, industry‐compatible fabrication processes. While lead‐free alternatives based on group IVA elements such as tin have shown encouraging optoelectronic properties, their widespread adoption is limited by their chemical instability under ambient conditions. Antimony‐based perovskite analogues are an emerging robust alternative, offering enhanced environmental stability, yet their synthesis has thus far depended on low‐throughput single‐crystal growth or solution processing routes that involve hazardous solvents and offer limited control over film structure and uniformity. Here, we demonstrate the first wafer‐scale, room‐temperature synthesis of high‐quality lead‐free Cs_3_Sb_2_Br_9_ and Cs_3_Sb_2_I_9_ thin films using radio frequency magnetron sputtering, a scalable, solvent‐free deposition technique extensively employed in semiconductor and display manufacturing for the large‐area deposition of metals, transparent conductive oxides and dielectric layers. The resulting films exhibit single‐phase crystallinity, tuneable optical bandgap ranging from 2.17 to 2.71 eV, and are well suited for UV–visible optoelectronics. Integrated into planar photodetectors, these materials achieve a photoresponsivity of 3.3 A/W, a bandwidth of 11 kHz, a linear dynamic range of 165 dB, and a detectivity of 1.7 × 10^15^ Jones, surpassing many state‐of‐the‐art devices. These findings establish magnetron sputtering as a powerful platform for the scalable fabrication of lead‐free perovskite optoelectronics and lay the foundation for their application beyond photovoltaics.

## Graphical abstract

We present the first wafer‐scale, room‐temperature synthesis of lead‐free Cs_3_Sb_2_X_9_ (X = Br, I) perovskite films via solvent‐free magnetron sputtering. The resulting high‐performance photodetectors achieve benchmark responsivity, bandwidth, and detectivity, enabling scalable manufacturing for next‐generation optoelectronics.

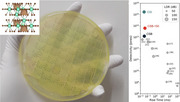

## 1 Introduction

Metal halide perovskites have emerged as one of the most versatile classes of semiconductors in modern materials science, unlocking unprecedented advances across optoelectronics, including solar cells [1], lighting technologies [2], photodetectors for imaging [3] and x‐ray sensing. Their exceptional tunability, low‐temperature processability, and remarkable photophysica properties such as strong light absorption, long photoexcited carrier diffusion length and defect states engineering, have placed them at the forefront of next‐generation neuromorphic plasticity [4] and computing [5], healthcare and environmental sensing technologies. Despite rapid progress, the transition of perovskite‐based devices from lab‐scale prototypes to industrially viable platforms remains hindered by two critical challenges: the widespread reliance on toxic lead‐containing compositions and the absence of suitable scalable and sustainable manufacturing strategies. Whilst significant steps forward in the thermal deposition of lead‐based materials are garnering widespread attention, the end‐of‐life disposal of lead‐based perovskite solar panels present major ecological and regulatory challenges as demonstrated through the leaching of lead from disposed solar panels into plants [6]. Consequently, lead‐free perovskites are being actively explored as a sustainable alternative [7, 8, 9, 10, 11, 12, 13]. However, beyond eliminating toxicity, these materials must also address critical challenges, including air stability and cost‐effective synthesis compatible with large‐scale manufacturing without compromising their optoelectronic properties [14].

Meeting these stringent requirements is particularly challenging due to the limitations imposed by the Goldschmidt tolerance factor, which restricts the range of suitable metal substitutions. Group IVA elements, particularly Sn, have emerged as potential alternatives owing to their electronic similarity to Pb. Among them, Sn‐based perovskites exhibit promising optoelectronic properties, yet their practical application is hindered by the instability of Sn^2 +^, which readily oxidizes to Sn^4 +^ under ambient conditions, leading to rapid degradation [15]. In contrast, perovskite‐analogues based on group VA elements, particularly Sb, offer enhanced environmental stability, making them viable candidates for Pb‐free photodetectors [16]. To date, the best‐performing Sb‐based photodetectors have been fabricated using single crystals, benefiting from their low defect density and superior charge transport properties [17, 18, 19, 20, 21]. However, the limited scalability of solvothermal or inverse crystallization methods for the synthesis of high‐quality single crystals hinders the broader commercial adoption of Sb‐based perovskites in device technologies.

Recent advances in solution‐processed Sb‐based perovskite‐like polycrystalline films have demonstrated high photoresponsivity, detectivity, and fast response times in photodetectors [22]. These findings underscore the crucial role of high‐quality polycrystalline films in achieving cost‐effective, scalable production of lead‐free perovskites for next‐generation optoelectronic devices. While solution processing is widely regarded as a low‐cost fabrication route, it often relies on toxic solvents such as dimethylformamide, thereby shifting the environmental burden to the synthesis stage. Furthermore, solution‐grown films frequently suffer from unintentional impurities and suboptimal crystallinity, which can adversely impact their optoelectronic properties. Solvent‐free deposition techniques offer a promising path to overcome these limitations while enabling the fabrication of high‐quality, environmentally sustainable perovskite films. Among these, chemical vapor deposition (CVD) has been explored in the synthesis of Cs_3_Sb_2_X_9_ (X = Br, I). However, the disparate melting temperatures of the precursors require a multi‐step process with elevated growth temperatures (⩾350 °C), restricting the range of compatible substrates [23]. Radio frequency (RF) magnetron sputtering of hybrid lead halide perovskites is an emerging compelling alternative pathway to the low‐temperature synthesis of perovskites offering precise stoichiometric control without the need for precursor melting or evaporation [24, 25]. Presently, the potential of this technique for the synthesis of Sb‐based perovskites remains largely unexplored, presenting an opportunity to develop scalable, high‐performance lead‐free optoelectronic materials.

Here, we pioneer the room temperature wafer‐scale growth of lead‐free perovskite analogues based on antimony by RF magnetron sputtering. In addition to being compatible with a wide range of substrates and viable for large‐scale manufacturing, our synthesis method offers a straightforward solution to vary the composition of the perovskite by changing the halide elements. Hence, we show that the quality of the synthesized materials supports the wafer‐scale manufacturing of Sb‐perovskite‐analogue photodetectors with a photoresponsivity of 3.3 A/W, bandwidth of 11 kHz, and a detectivity of 1.7× 10^15^ Jones, surpassing the performances reported in most of the state‐of‐the‐art single‐crystal based devices [17, 18]. The reduced density of impurities and the high stability of the thin film produced with our method underpin the fabrication of photodetectors with an extraordinarily large linear dynamic range of 165 dB and stable levels of photocurrent measured while keeping the devices in air for at least 20 hours without any encapsulation. Our findings are a stepping stone to the wider development of lead‐free and sustainable perovskites with a unique potential for underpinning applications beyond photovoltaics.

## 2 Results and Discussion

**FIGURE 1:**
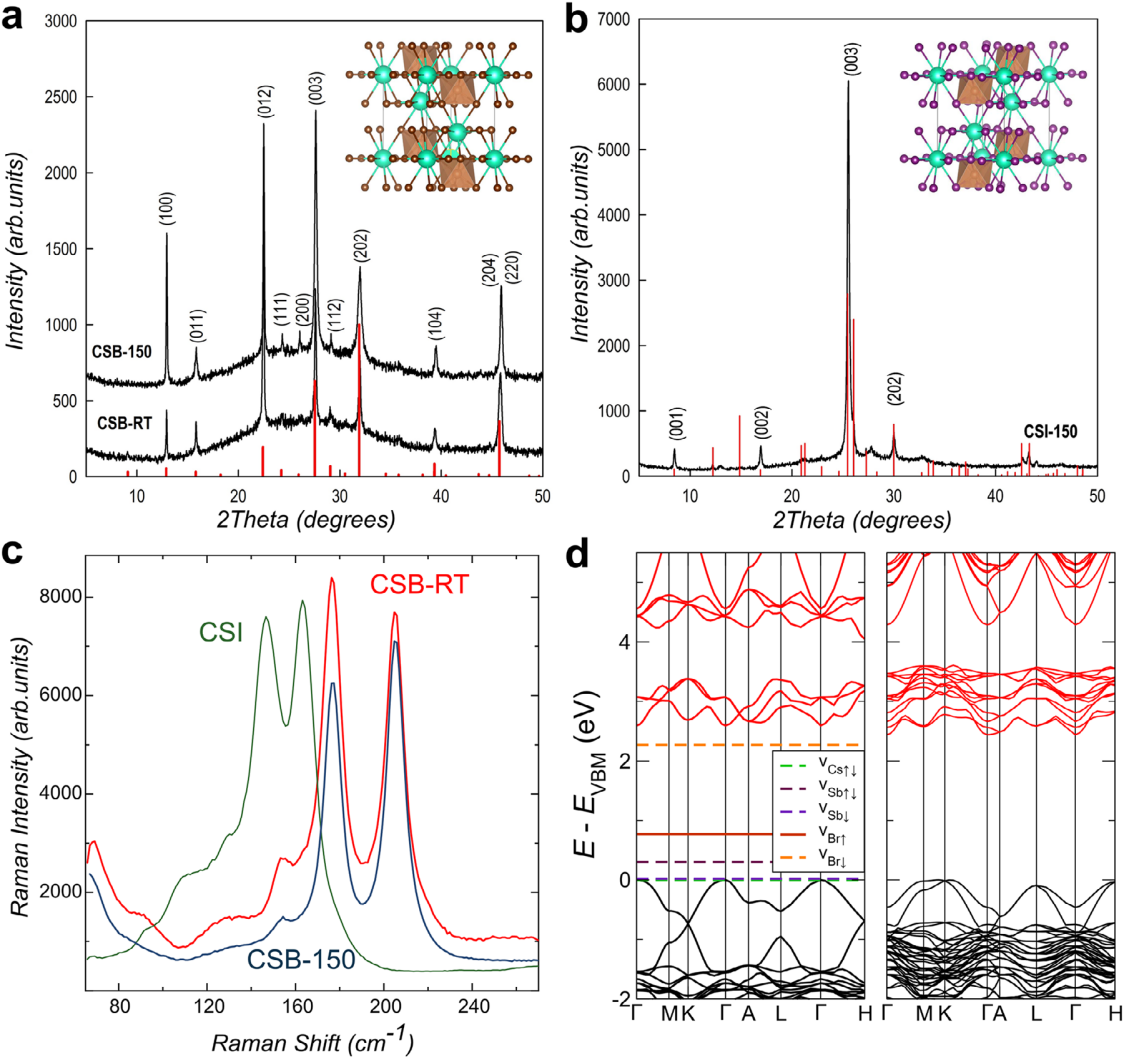
(a,b) Plots of the XRD measurements for CSB deposited at room temperature and 150°C (black curves in (a)), and for CSI (black curves in (b)). Red bars indicate expected peak positions for the corresponding crystal structures shown in the insets. (c) Raman spectra of CSB‐RT (red), CSB‐150 (blue) and CSI (green), see Methods. (d) Electronic band structures of CSB (left) and CSI (right), obtained from DFT calculations using the HSE06 functional. For CSB, the plot includes energy levels associated with various vacancies, with spin polarization taken into account. Bold lines represent occupied states, while dashed lines indicate unoccupied states. The simulated band structures reveal a direct bandgap of 2.59 eV for CSB and 2.44 eV for CSI.

Thin films of Cs_3_Sb_2_Br_9_ and Cs_3_Sb_2_I_9_ (300 ± 10 nm thick) were grown by RF‐magnetron sputtering starting from stoichiometric targets obtained following the procedure described in Supporting Information S1. The use of room‐temperature magnetron sputtering squarely aligns with industrial manufacturing paradigms, enabling solvent‐free, uniform, and scalable deposition over large substrates. This mature, industry‐established thin‐film technology is already adopted by the semiconductor manufacturing industry at wafer and meter scales for example in flat‐panel displays and photovoltaics manufacturing, supporting its potential scalability for large‐area perovskite deposition. X‐ray diffraction (XRD) was used to identify the crystallinity of the films and the optimal deposition parameters for the growth of a single phase, see Methods. Figure 1a shows representative XRD spectra measured on Cs_3_Sb_2_Br_9_ films deposited on substrates maintained at room temperature (CSB‐RT) and 150 °C (CSB‐150). The observed peaks are in agreement with the hexagonal space group of $P\bar{3}m1$ (reported as vertical red bars) and unit cell *a* = *b* = 7.93 Å, *c* = 9.716 Å, α = β = 90°, γ = 120°, expected for this type of perovskite [17]. The similarities in the widths and positions of the XRD peaks for the films of CSB‐RT and CSB‐150 demonstrate that the explored range of growth temperature results in films with analogous structure. Furthermore, by comparing the experimental and expected peak intensities, it is possible to rule out any relevant preferential orientation effect. Finally, the Rietvald analysis of the XRD data reveals that the ratio of the (100):(011) peak intensities is heavily influenced by the Sb:Cs ratio, indicating different atomic fractions of Cs for CSB‐150 and CSB‐RT likely to underpin a different density of defect states.

Figure 1b shows the XRD spectrum for a Cs_3_Sb_2_I_9_ film grown on a heated substrate at 150 °C (CSI), compared with the expected diffraction pattern of the layered polymorph (vertical red bars), which crystallizes in the $P\bar{3}m1$ space group with lattice parameters *a* = *b* = 8.435 Å, *c* = 10.390 Å, α = β = 90°, γ = 120°. Unlike in the CSB case, the CSI film exhibits a pronounced preferential orientation effect, particularly along the (*h*0*l*) and (00*l*) planes. The presence of the (002), (004), and (006) diffraction peaks further confirms a well‐defined periodicity along the c‐axis, consistent with the layered structure composed of corner‐sharing SbI_6_ octahedra separated by Cs cations. This structural characterization confirms that RF‐magnetron sputtering enables the direct synthesis of the desired $P\bar{3}m1$ phase without the use of additional solvents or any rigorous post‐synthesis annealing such as the case for solution‐based methods. At the same time, the dominant XRD peaks in CSI‐150 suggest strong orientation along the [001] direction, while the CSB films exhibit a more randomly oriented polycrystalline texture, possibly due to the halide‐dependent crystallization kinetics and substrate interaction effects, where the iodide promotes highly oriented growth under the given synthesis conditions.

To gain further insight into the structure of the films we employ vibrational spectroscopy. Figure 1c shows the room temperature Raman spectra for CSB‐RT, CSB‐150 and CSI thin films. The factor group analysis of the 14‐atom $P\bar{3}m1$ (D3d) structure predicts 9 Raman‐active modes encompassing the symmetric (4A_1*g*_) and asymmetric (5E_*g*_) stretching of bonds. [26] Therefore, the Raman spectra of these perovskite derivatives are dominated by the activity of the octahedral cages, i.e., SbBr_6_ and SbI_6_. Our experimental data are in good agreement with previous studies [27] on single crystals reporting, the A_1*g*_ peak at 164.8 cm^−1^ and the Eg at 146.8 cm^−1^ stemming from the Sb‐I displacements inside the octahedra. For CSB‐RT and CSB‐150, these Raman peaks appear at higher energies with the A_1*g*_ at 204.7 cm^−1^ and the E_*g*_ at 176.3 cm^−1^, consistent with theoretically predicted values of 203.5 cm^−1^ and 176.2 cm^−1^, respectively [28]. The Raman spectra of both the CSB and CSI samples display additional peaks in the range of 80–135 cm^−1^ with intensities smaller than A_1*g*_ and E_*g*_, which can be attributed to bending modes, lattice deformation modes, or a combination of both. To gain deeper insight into the electronic behavior of the material, we performed first‐principles band structure calculations using density functional theory (DFT) with the HSE06 hybrid functional, as implemented in VASP. The resulting band structures, presented in Figure 1d and discussed in detail in Section S1 of the Supporting Information, reveal distinct features for the two polymorphs. The left panel corresponds to the CSB phase, for which we also computed defect formation energies using the GGA‐PBE functional to explore the impact of vacancy configurations. In contrast, the right panel shows the electronic structure of the CSI phase, highlighting key differences in their band dispersion and electronic character.

**FIGURE 2:**
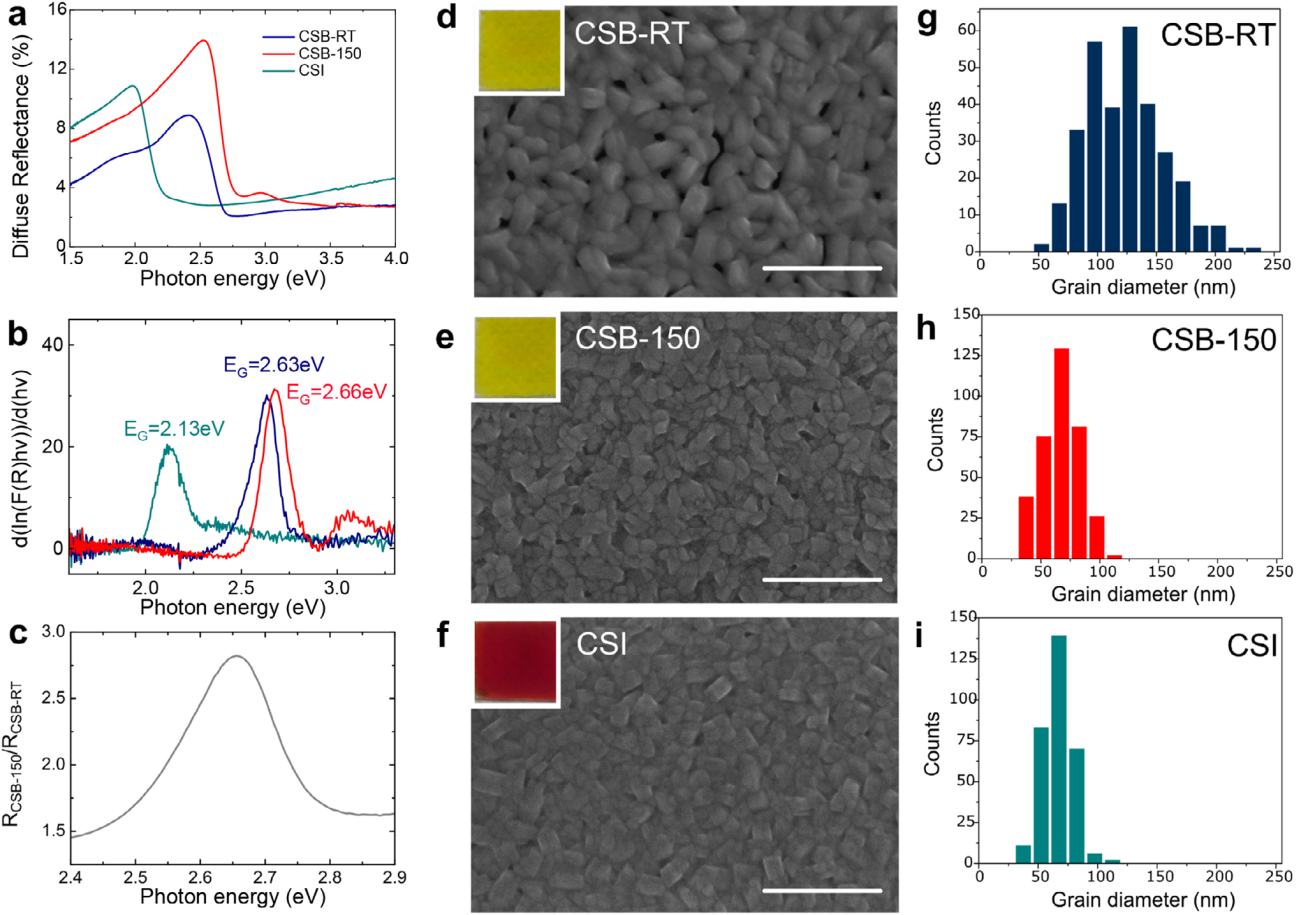
(a) Measured spectral diffuse reflectance of the sputtered lead‐free perovskite thin films. (b) Plot of $\frac{d(\ln(F(R)h\nu ))}{d(h\nu )}$ versus *h*ν for the three types of perovksites and corresponding values of the energy gap. (c) Plot of the diffuse reflectance ratio *R*_*CSB* − 150_/*R*_*CSB* − *RT*_ versus photon energy. (d–f) SEM images of the sputtered lead‐free perovskites. The white scale bar corresponds to 500 nm. Insets: white‐light photographs of the relative films deposited on quartz. (g–i) Frequency plots of the perovskite grain sizes obtained from the analysis of the SEM images shown in panels (d–f).

The experimental study of the spectral response of the optical diffuse reflectance (*R*) and absorbance enable the validation of the theoretically predicted values for the semiconducting energy gap (*E*_*gap*_) of CSB and CSI perovskites, see Figure 2a and Figure S2 (Supporting Information). The optical bandgap was estimated using the Tauc method, which relates the absorption coefficient α to the photon energy *h*ν through the expression (α*h*ν)^1/γ^ = *B*(*h*ν − *E*_*gap*_) where *B* is a constant and γ = 1/2 or 2 for direct or indirect optical transitions, respectively [29] At the same time, diffuse reflectance spectroscopy provides an independent way to determine the energy gap through the Kubelka‐Munk function *F*(*R*) = (1 − *R*)^2^/2*R* and its dependence on the photon energy and energy gap (*F*(*R*)*h*ν)^1/γ^ = *B*(*h*ν − *E*_*gap*_). [30] This results in a turning point near the absorption edge of a direct gap semiconductor $\frac{d(\ln F(R)h\nu )}{d(h\nu )}=\frac{1}{2(h\nu -E_{gap})}$. Figure 2b shows that this analysis on the experimental data gives *E*_*gap*_ = 2.13 eV (CSI), 2.63 eV (CSB‐RT) and 2.66 eV (CSB‐150), consistent with predicted values and prior studies [31, 32] albeit the use of HSE06 over PBE in DFT is known to lead to slightly overestimated values of the energy gap as in the case of CSI [33, 34]. Additionally, the energy‐resolved ratio *R*_*CSB* − 150_/*R*_*CSB* − *RT*_ reaches a maximum value at ≈5 meV above the band edge of CSB‐RT (see Figure 2c), confirming the distinct values of *E*_*gap*_ for the room‐temperature and 150 °C synthesized films, despite their identical chemical and structural composition [35].

Scanning electron images for the studied perovskites sputtered on quartz reveal a clear difference in the grain size distribution, with an average grain size 50 nm smaller for the CSB‐150 than for CSB‐RT consistent with a large density of nucleation sites for high substrate temperature. Such variance in the grain size could be expected to influence the percolation topology of conductive pathways with smaller grains providing a larger number of parallel conduction paths. At the same time, the relative difference of average grain size values excludes a possible role of quantum confinement on the origin of the observed energy gap difference, as this would result in a shift of less than 1 meV. The polycrystalline nature of the films and their XRD data analysis suggest that defects could play a key role on the properties of these materials. Indeed, defect‐induced sub‐gap states can facilitate optical absorption and emission of photons at energies lower than the intrinsic bandgap, i.e., Urbach tail. To this end, perovskites of identical chemical composition but differing average grain sizes, such as the case for CSB, are ideally suited to reveal whether surface or bulk defects are predominantly responsible for the band edge tails. Within this framework, the energy‐resolved reflectance ratio for CSB‐RT to CSB‐150 is expected to bear direct information on the ratio of the defect states, reaching a maximum value of 2.8, see Figure 2c. The corresponding analysis of the average measured grain surface and volume ratio reveals values of ≈3 and 6, respectively (see morphological analysis in Materials and Methods), demonstrating that defects on the surface of the grains are responsible for the smaller energy gap observed in CSB‐RT compared to CSB‐150. First‐principles calculations offer a further insight on the nature of these defects. To this end, we have considered the three principle vacancies such as Cs, Sb, and Br, see Supporting Information. We find that Cs and Sb vacancies introduce a light p‐type doping near the band edge of CSB, whereas the Br vacancy introduces two deep states at 0.8 and 2.3 eV. Interestingly, the relative ratios of these defects change from the bulk to the surface. More specifically, the formation energy of Br vacancies remains relatively unaffected as to whether they are located on the surface or in the bulk, whereas Cs and Sb vacancies become more likely on the surface. This is consistent with the changes in atomic fractions for CSB‐RT and CSB‐150 observed in the XRD measurements. Therefore, shallow p‐type doping due to surface defect states is expected to play a key role in the photophysical properties of CSB.

**FIGURE 3:**
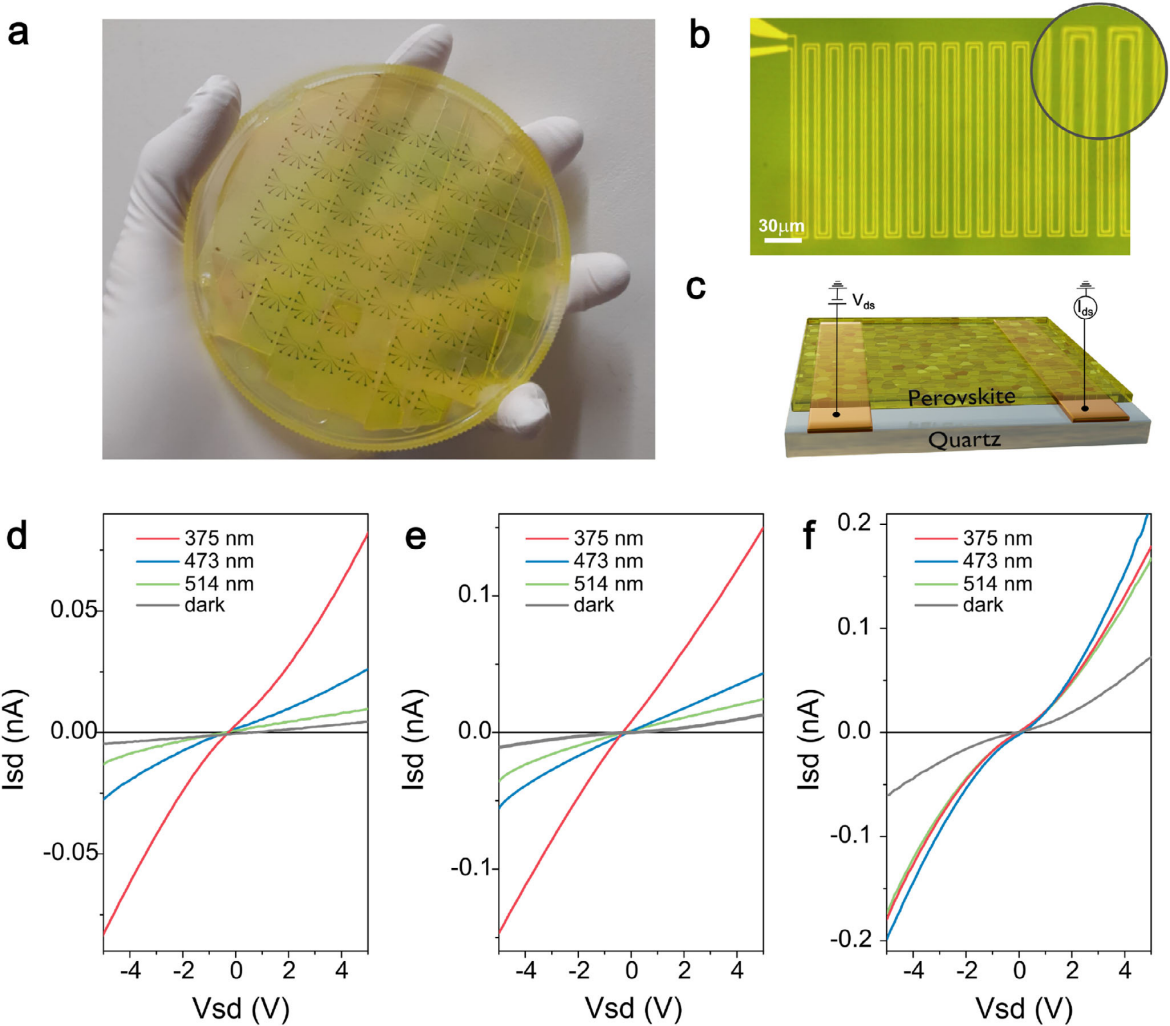
(a) Photograph showing the 4‐inch wafer scale fabrication of CSB photodetectors on a quartz substrate. (b) Optical micrograph showing a close‐up detail of the serpentine design adopted for the electrical contacts of individual photodetectors, with a source‐drain channel length of 3 µm. (c) Schematic of the photodetector structure and electrical circuit configuration used to characterize their performance. The device consists of a sputtered perovskite film deposited onto pre‐patterned contacts. (d–f) Plots of the measured source‐drain current characteristics of CSB‐RT (d), CSB‐150 (e), and CSI (f) in dark and under illumination with CW lasers as specified in the legend, see Methods for details on irradiance and photoactive areas.

The wafer‐scale magnetron sputtering deposition of single phase lead‐free perovskites is cornerstone to underpinning a wider technological adotpion of these materials. To this end, we have processed wafer scale, 4‐inches diameter, photodetectors based on sputtered films and characterized their figures of merit, see Methods and Figure 3a–c. The interdigitated metal electrodes and short channel length result in a large aspect ratio of the semiconducting channel (width/length≫1) supporting large values of photoactive surface area and photocurrent with photo‐generated carriers traversing a minimal distance before collection. Figure 3d–f shows plots of the measured photocurrent in representative photodetectors of each type of perovskite in dark conditions and upon illuminating with continuous wave laser beams of different wavelengths (375, 473, and 514 nm). Fixed source‐drain bias of 5 V for CSB‐RT and CSB‐150 and 10 V for CSI were used due to the different levels of dark current and device resistance. In all cases a photocurrent is measured throughout the perovskite channels independently of the laser spot position, demonstrating the efficient extraction of photo‐excited carriers from the semiconducting channel. The devices remain stable under ambient conditions even after 68 hours in air, with a noticeable increase in photocurrent following air exposure, likely due to oxygen passivation [34]. Additionally, the photocurrent measured from chips across the wafer exhibits highly reproducible values, corresponding to a device‐to‐device spread of less than 14%, see Supporting Information S4.

**FIGURE 4:**
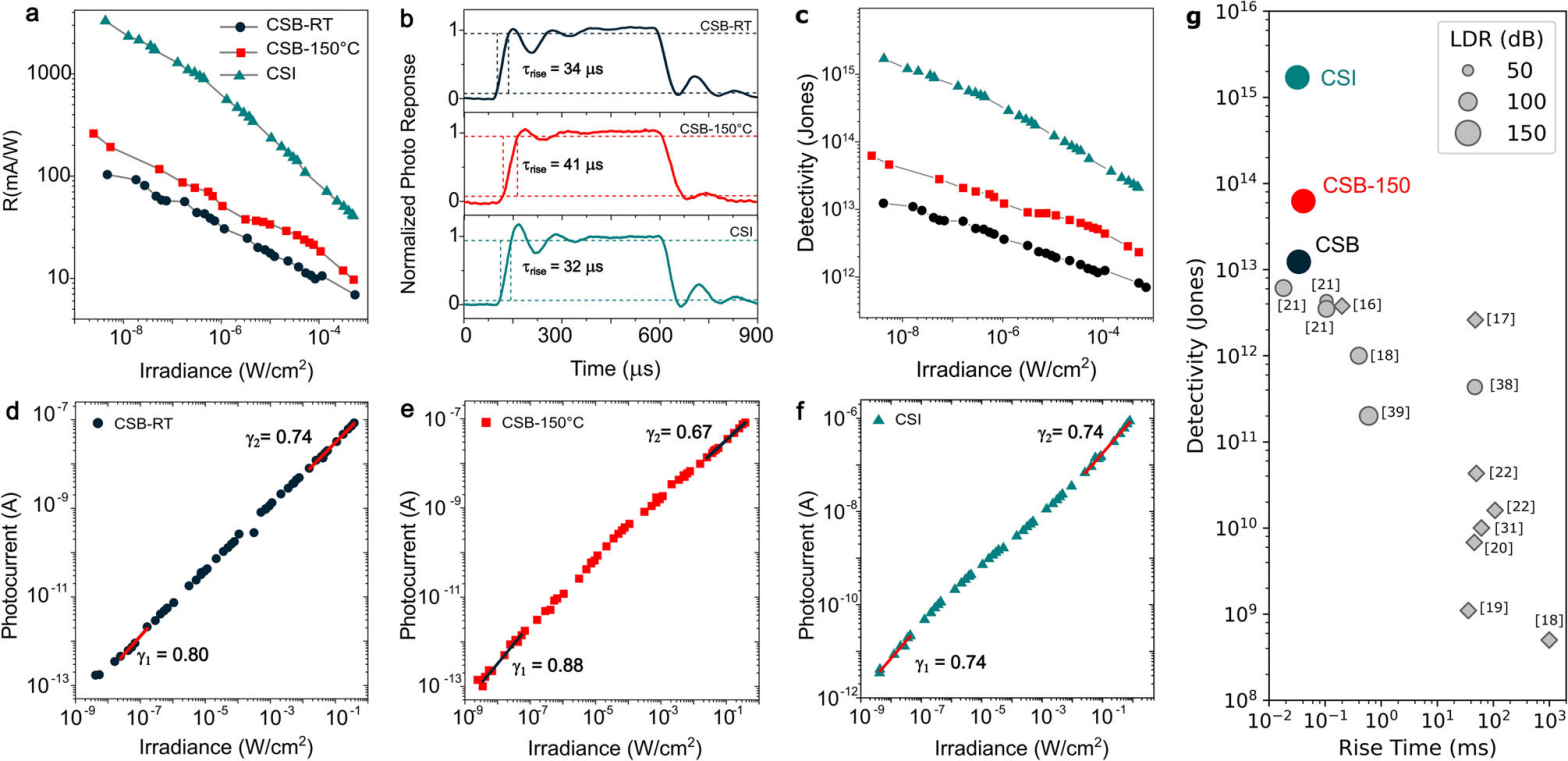
(a) Plots of the measured power dependent photoresponsivity for CSB‐RT, CSB‐150, and CSI photodetectors at V_*SD*_ = 10 V and under uniform illumination (λ = 375 nm for CSB and 473 nm for CSI). (b) Time response of the devices in (a) for constant Vsd = 15 V (λ = 375 nm for CSB an 473 nm for CSI). (c) Plot of the photodetectivity for the devices in (a). (d–f) Plot of the linear dynamic range (LDR) measured at a fixed V_*SD*_ = 10 V for the devices in (a). (g) Plot of detectivity versus rise time of state‐of‐the‐art lead‐free based photodetectors compared to the measured values in this work. All the measurements have been performed by illuminating the CSB and CSI based devices with a 375 nm and a 473 nm laser, respectively.

Figure 4a shows a plot of the room‐temperature photoresponsivity (R) measured in CSB and CSI photodetectors in vacuum as a function of irradiance with photon wavelengths matching the bandgap energy (CSB with 375 nm and CSI with 473 nm for CSI) and for a constant *V*_*SD*_ = 10 V, see Methods. The highest values of R were recorded at ultra‐low irradiance levels, reaching 110 mA/W for CSB‐RT at 4.19 × 10^−9^ W/cm^2^, 261 mA/W for CSB‐150 at 2.44 × 10^−9^ W/cm^2^, and an exceptionally high 3294 mA/W for CSI at 4.3 × 10^−9^ W/cm^2^. These figures of merit rival some of the best values reported to date in other Sb‐based perovskites, see Table S2. Furthermore, as the incident optical power increases R decreases monotonously, indicating that traps become saturated, reducing their ability to contribute to the photocurrent [36]. Figure 4b shows the time response of the photocurrent measured by illuminating the photodetectors with laser pulses of approximately 450 µs duration (λ = 375 nm for CSB and 473 nm for CSI at 4 × 10^−2^ W/cm^2^) while maintaining a fixed *V*_*SD*_ = 15 V, see Methods. The measured rise times are 34µs for CSB‐RT, 41µs for CSB‐150, and 32µs for CSI, corresponding to a bandwidth of ≈ 11 kHz. Hence, these wafer‐scale 3 µm channel planar photodetectors are highly suitable for a range of applications to include optical audio transfer, that is transmission of audio signals via light with commercial mobile phone voice quality requiring <10 KHz. In these devices the time response is critically influenced by the photogenerated carrier transit time [37, 38] (*t*_*tr*_) which has a quadratic dependence on the channel length (*t*_*tr*_ = *L* ^2^/µ*V*_*SD*_). Hence, the down‐scaling of the semiconducting channel to 0.1 µm can potentially result in devices with ≈10 MHz bandwidth provided that electrical transport is drift‐dominated and the devices are not operated in the high‐field velocity‐saturation regime, see Supporting Information. This provides a pathway to engineer the device performance through the design of the contacts, and exploit the potential of these lead‐free perovskites for a wide range of optoelectornic applications from imaging to communication. Finally, the low levels of noise spectral density and large photoresponsivity measured in these devices result in high specific detectivity values (D*) of 1.23 × 10^13^ Jones for CSB‐RT, 6.23 × 10^13^ Jones for CSB‐150, and a staggering 1.70 × 10^15^ Jones for CSI at the lowest values of irradiance and considering the largest measured values of noise spectral density, see Figure 4c.

Figure 4d–f presents the irradiance‐dependent photocurrent response for the three perovskite samples on a log–log scale, covering an exceptionally wide dynamic range: 163.9 dB for CSB‐RT and CSB‐150, and 165.4 dB for CSI. Each shows a near‐linear dependence across this range, significantly exceeding the dynamic ranges reported for Sb‐based perovskites (see Figure 4g) [19, 22, 39, 40]. The power‐law scaling of the photocurrent (*I*_*ph*_) with irradiance (*P*), *I*_*ph*_∝*P* ^γ^, provides insight into the underlying recombination mechanisms through the exponent γ [41, 42]. Typically at low irradiance, trap‐assisted recombination dominates, often manifesting as monomolecular behavior with γ ≈ 1 whereas non linear behavior is observed at high irradiance due to the emergence of space charge effects [43] and/or bimolecular recombination with γ = 0.5 [44, 45, 46]. At the same time, depending on the energy depth and concentration of trap states non‐linearities are also expected at low light intensities when changes in trap occupation occur [45]. Deep traps, which are largely unaffected by illumination level, produce linear photocurrent scaling (γ ≈ 1), while shallow traps associated with Urbach tail states cause a reduced slope that depends on the characteristic energy of the trap distribution [47]. At intermediate irradiance, shallow traps may become partially occupied and behave as deeper traps, leading to an “S‐shaped” photocurrent response, where the transition point depends on trap energy depth and bandgap width [44, 47]. Among the materials studied, CSB‐150 exhibits nearly linear scaling at low irradiance γ ≈ 0.9, consistent with the dominance of deep traps. In contrast, CSB‐RT (γ = 0.82) and CSI (γ = 0.74) show stronger deviations, indicative of shallower trap states. This trend is supported by Urbach energy measurements revealing *E*_*U*_ ≈ 140*meV* for CSB‐150, *E*_*U*_ ≈ 120*meV* for CSI and *E*_*U*_ ≈ 100*meV* CSB‐RT (see S2 in the Supporting Information). At higher irradiance, CSB‐150 and CSB‐RT exhibit a transition toward bimolecular recombination, reflected by γ = 0.5, while CSI maintains γ = 0.74 across the full irradiance range. This persistent sublinear behavior, along with the observed S‐shaped response, suggests a mixed recombination regime in CSI, likely involving the gradual filling of shallow trap states [44, 45].

## 3 Conclusion

In conclusion, in this work we have demonstrated the first wafer‐scale synthesis of lead‐free perovskite analogues based on antimony using magnetron sputtering at room temperature, a scalable and industry‐compatible method that enables precise stoichiometric control and improved crystallinity. The resulting Cs_3_Sb_2_Br_9_ and Cs_3_Sb_2_I_9_ thin films exhibit a single‐phase crystalline structure, as confirmed by X‐ray diffraction and vibrational spectroscopy, with optical bandgap spanning 2.17–2.71 eV, well suited for UV–visible optoelectronics. Integrated into planar photodetectors, these materials yield exceptional performance metrics, including a photoresponsivity of 3.3 A/W, a bandwidth of 11 kHz, a linear dynamic range of 165 dB, and a detectivity of 1.7 × 10^15^ Jones, surpassing many single‐crystal‐based devices. The further down‐scaling of the photodetectors channel to 0.1 µm has the potential to boost the bandwidth of the devices to 10 MHz in the transit‐time‐limited transport, making them ideally suited to a wide range of optoelectronic applications from optical audio and video transfer to sensing. These findings establish magnetron sputtering as a compelling platform for the scalable room‐temperature deposition of high‐quality lead‐free perovskite thin films. More broadly, this work advances the development of sustainable optoelectronic perovskite platforms beyond photovoltaics, with potential applications in visible light communication, smart imaging, environmental monitoring, biomedical sensing, and integrated photonics for next‐generation wearable and Internet of Things technologies by improving the spectral bandwidth leveraging complementary strategies such as halide alloying [48, 49], Schottky‐barrier engineering [50, 51], and heterostructure integration with narrower‐bandgap materials [52, 53].

## 4 Materials and Methods

### 4.1 Thin Film Preparation

*Cs*_3_ *Sb*_2_ *Br*_9_ and *Cs*_3_ *Sb*_2_ *I*_9_ films have been deposited by RF magnetron sputtering starting from a single target consisting in a mixture of stoichiometric quantities of CsI(CsBr), SbI_3_, and SbBr_3_. The sputtering conditions were: power of 50 W, argon flux of 20 SCCM, pressure in the chamber of 0.023 mbar, and 10 cm target‐substrate distance. Depositions of 300 nm thick films have been carried out both without any heating and by heating the substrate during the deposition at 150°C. The thickness value of the samples has been determined by a stylus profilometer P‐7 KLA Tencor applying a force of 2 mg with a silicon stylus of 2 μm radius and using a Bruker Innova Atomic Force Microscope.

### 4.2 Wafer‐Scale Fabrication of Photodetectors

Interdigitated metal electrodes were fabricated on 4‐inch quartz wafers by standard electron‐beam lithography, metal deposition, and lift‐off following previousely established procedures [54]. Briefly, prior to patterning, the wafer was spin‐coated with a ≈600 nm thick layer of 495K A6 PMMA resist and water‐soluble AR‐PC 5090 conductive protective coating, each baked for 1 min at 180°C. The design of the interdigitated electrodes and bonding pads was written by electron‐beam lithography with a dose of 1000 µC/m^2^. Before the development step, the AR‐PC 5090 was removed by rinsing in de‐ionised water, and the lithorgraphically defined pattern was revealed by developing the PMMA for 30 s in a solution of IPA:MIBK:MEK (15:5:1 by volume) followed by a rinse in IPA for 45 s. Metal contacts were then deposited by high‐vacuum electron‐beam evaporation (base pressure ∼2 × 10^−8^ mbar), consisting of a Ti adhesion layer (5nm) and an Au overlayer (50nm). The lift‐off process was carried out by soaking the wafer in warm acetone (70°C) for at least 2 hours, removing any redundant metal deposited on PMMA, and leaving the well‐defined interdigitated electrodes. The perovskites films were deposited by vapour phase onto the whole wafer following the procedures described in the Thin Film Preparation of the Methods section. Hence, individual chips of 1 × 1cm^2^, each comprising 3 photodetectors, were diced and characterised in a home developed opto‐electronic characterization tool with integrated spectroscopic and opto‐electronic characterization capabilities [4].

### 4.3 Raman Spectroscopy

Micro‐Raman measurements were carried out at room temperature in ambient conditions by using a Labram Dilor spectrometer equipped with an Olympus microscope HS BX40. The 632.8 nm light from He‐Ne laser was employed as excitation radiation. The samples, mounted on a motorized xy stage, were tested with a 50× objective and with a laser spot of about 2 mm in diameter. The spectral resolution was about 1 cm^−1^. The reported spectra are obtained as the average over 20 spectra acquired on the film surface, providing direct evidence for phase homogeneity.

### 4.4 Optical Spectroscopy

Ultraviolet‐Visible‐Near Infrared (UV‐Vis‐NIR) optical measurements were performed under ambient conditions using a Varian Cary 6000i spectrophotometer equipped with a double monochromator, a deuterium lamp and a tungsten filament lamp as light sources, a Si photomultiplier (UV‐Vis) and an InGaAs photodiode (NIR) as detectors. Spectral range was 200–1800 nm, in steps of 1 nm. Both near normal absorption spectra and diffuse reflectance spectra with a 110 mm diameter integrating sphere were measured.

### 4.5 Morphological Characterization

Sputtered films on quartz substrates where imaged with a scanning electron microscope employing a low acceleration voltage of 10 kV and an emission current of 0.13 µA. The average grain size diameter measured for CSB‐RT is 120 nm, corresponding to a volume of ≈1 × 10^−21^ m^3^ and a surface area of ≈1.54 × 10^−13^ m^2^. For CSB‐150 the average grain diameter is 70 nm, resulting in a volume of ≈1.79 × 10^−22^ m^3^ and a surface area of ≈4.83 × 10^−14^ m^2^.

### 4.6 Optoelectronic Characterization

An **integrated custom‐built opto‐electronic setup** is used for the characterization of perovskites [4]. Briefly, this consists of a number of light sources (to include continuous wave and pulsed lasers in the UV‐Vis wavelength range and calibrated white light) interfaced to an upright microscope. The home developed vacuum chamber with electrical lines and optically transparent windows is mounted on a motorized ProScan III microscope stage. Finally a suitable spectrometer enables the *in* − *situ* characterization of spatially resolved Raman [55], photoluminescence and optical absorption, transmission and absorption measurements, as well as spatially resolved photocurrent [56]. The data shown in Figure 3d–f correspond to a photoactive area of 2.2 × 10^−4^m^2^ when illuminating with the 375 nm CW laser, and 2.9 × 10^−4^m^2^ for the 472 and 514 nm with the slight difference due to the different optical paths and elements used for the different wavelengths.

**Real time photocurrent** signals were acquired using a real‐time digital oscilloscope (Rohde & Schwarz 2 GHz Series 1000 Digital Oscilloscope, 2 Gbit/s sampling rate) and a 50 Ω input termination, whereas the triggering of the laser sources (λ = 473 nm, laser rise‐ and fall‐time <50 ns) was carried out by using a square wave signal generated by a Tektronix AFG1022 Arbitrary Waveform Generator [56]. The spectrally‐resolved photocurrent measurements were carried out using a 300 W xenon lamp source coupled to a monocromator with 10 nm resolution (Newport TLS300X).

The **photoresponsivity** is defined as the ratio of the generated photocurrent to the incident optical power on the active area, where the generated photocurrent corresponds to the difference between the current measured in the devices under illumination minus the current measured while keeping the photodetectors in dark conditions [57].

## Author Contributions

Rosanna Mastria and Hoi Tung Lam have contributed equally to this work. RM and HTL conducted the fabrication, characterization and data analysis of the devices. IL analysed the linear dynamic range and Urbach energy. MP and SB has acquired and analysed the reflectance spectroscopy. PG and LM have synthesized the perovskites and characterized them by XRD and Raman spectroscopy. MASA has contributed to the fabrication of the interdigitated contacts. NT and SH have conducted the DFT simulations. LM, MFC and SR planned and directed the experiments and the interpretation of their results. All authors contributed to discussions, and the writing of the article.

## Conflicts of Interest

The authors declare no conflict of interest.

## Supporting information


**Supporting File**: advs73736‐sup‐0001‐SuppMat.pdf.

## Data Availability

The data that support the findings of this study are available from the corresponding author upon reasonable request.
