# Supplementary material for: Wafer‐Scale Room‐Temperature Processing of Lead‐Free Perovskites for Optoelectronic Applications
Source: Adv Sci (Weinh). 2026 Feb 9;13(16):e17469. doi: 10.1002/advs.202517469 (PMC13042632; doi:10.1002/advs.202517469)
Supplement: Supplementary file 1 — Supporting File: advs73736‐sup‐0001‐SuppMat.pdf. [file ADVS-13-e17469-s001.pdf]

Supporting Information: Wafer-Scale Room-Temperature Processing of Lead-Free Perovskites for Optoelectronic Applications

Rosanna Mastria,[†,#] Hoi Tung Lam,[†,#] Ioannis Leontis,[†] Sara Bonomi,[‡] Mohammed Ali Saleh Alshehri,[†] Aurora Rizzo,[¶] Pietro Galinetto,[§] Maddalena Patrini,[§] Ned Taylor,[†] Steven P. Hepplestone,[†] Monica F. Craciun,[‖] Lorenzo Malavasi,[*,⊥] and Saverio Russo[*,†]

†*Centre for Graphene Science, Department of Physics and Astronomy, University of Exeter, Stocker Road 6, EX4 4QL Exeter, United Kingdom*

‡*Department of Chemistry and INSTM, University of Pavia, Viale Taramelli 16, 27100 Pavia, Italy Pavia, Italy*

¶*CNR NANOTEC, Institute of Nanotechnology, via Monteroni, 73100, Lecce, Italy*

§*Department of Physics, University of Pavia, via Bassi 6, I-271 00 Pavia, Italy*

‖*Centre for Graphene Science, Department of Engineering, North Park Road, University of Exeter, EX4 4QF Exeter, United Kingdom*

⊥*Department of Chemistry and INSTM, University of Pavia, Viale Taramelli 16, 27100 Pavia, Italy*

#*These authors contributed equally to this work*

E-mail: lorenzo.malavasi@unipv.it; s.russo@exeter.ac.uk

S1. $Cs_3Sb_2Br_9$ defect formation energies

The formation energies, E_f of the various defects considered are calculated using the well established relation

$$E_f = E_D - E_0 + \mu_d \tag{1}$$

where E_D is the total energy of the system with the vacancy, E_0 is the energy of the same system without the vacancy and the μ_d is the chemical potential of the atom missing, creating the vacancy. For the values given in Table 1 we have chosen to set the chemical potential to the bulk species. As we are interested in the comparison between the surface and the bulk, the choice of chemical potential is relatively unimportant since defects are referenced to the same value.

In this work, first-principles techniques based on density functional theory (DFT) were used to determine the structural, electronic, and energetic properties of $Cs_3Sb_2Br_9$. These calculations were performed using the Vienna ab initio simulation package (VASP).[1,2] The valence electrons for each atomic species were considered as follows: Cs $5s^2$ $5p^6$ $6s^1$, Sb $5s^2$ $5p^3$, Br $4s^2$ $4p^5$. The projector augmented wave method was used to describe the interaction between core and valence electrons, and a plane-wave basis set was used with an energy cutoff of 600 eV. All calculations were completed using the Perdew-Burke-Ernzerhof (PBE) generalised gradient approximation functional,[3] and with spin-polarised settings.

Structures were relaxed using GGA-PBE. For geometric relaxations, all forces were relaxed to below 0.01 eV/Å per atom, with electronic self-consistency converged to 10^{-7} eV. Both atomic positions and lattice constants were allowed to relax for perfect and defected structures. In bulk, the structure is constrained by the surrounding material, but in slabs, this constraint is absent. Introducing a vacancy in a slab can cause compression or expansion along the surface normal due to the available vacuum. This allows strain compensation along the c-axis, a mechanism unavailable in bulk, impacting system energetics and result reliability. To ensure comparability, lattice constants were relaxed in both bulk and slab

vacancy calculations.

The energetics of the relaxed structures are obtained and analysed using GGA-PBE. The band structure was calculated by performing HSE06 hybrid functional[4] calculations on the GGA-PBE relaxed structures (commonly known as a *one-shot* approach), where HSE06 shows improved accuracy for band gaps and electronic properties.[5] All considerations of k-point grids are performed using the primitive cell as the reference, using $6\times6\times6$ Monkhorst-Pack grid.[6] Bulk vacancies are modelled in $2\times2\times2$ supercells, giving a vacancy concentration of 0.125 vac/unit (2.2×10^{20} cm^{-3}). For vacancies in slab structures (i.e. surface calculations), vacancies are modelled in 2×2 (extensions parallel to the surface plane) supercells, where the slab thickness is 2 unit cells thick.

Additionally, the formation energies of the vacancies calculated using GGA-PBE are compared to those obtained using the MACE-MP-0[7] machine learned potential (where the base model is used). This foundation model is chosen as it has shown good agreement with GGA-PBE for energetic properties of many perfect crystals. The MACE model results exhibit significant deviations from DFT calculations, both qualitatively and quantitatively. Not only do the predicted formation energies differ from those obtained via DFT, but the relative energetic ordering of defects is not achieved. This latter discrepancy is particularly concerning, as it suggests that the MACE foundation model fails to capture the correct energetic hierarchy within this chemical space. The inability to preserve the relative stability of defects indicates that MACE, on its own, is not a reliable energy calculator for this system and requires further fine tuning to capture the system correctly.

Table 1: The formation energies of neutral vacancies in the bulk and at the surface of $Cs_3Sb_2Br_9$ calculated with respect to the bulk chemical potentials using MACE-MP-0 and the PBE-GGA functional.

Defect	System Type	Formation energy PBE (eV)	Formation energy MACE (eV)
Cs vacancy	Bulk	4.0	3.0
Sb vacancy	Bulk	4.2	1.5
Br vacancy	Bulk	2.1	1.1
Cs vacancy	Surface	3.0	3.3
Sb vacancy	Surface	3.2	1.5
Br vacancy	Surface	2.2	1.4

S2. Analysis of diffuse reflectance and Urbach energy

The Urbach energy (E_U) characterizes the exponential tail of the absorption edge in semiconductors and insulators. This arises from disorder-induced band tail states, originating from structural disorder, phonon interactions, and defect states. In this study, we extract the Urbach energy from spectrally resolved diffuse reflectance (R) measurements of lead-free perovskites. The absorption edge is determined using the Kubelka-Munk function ($F(R)$), allowing us to analyze the low-energy exponential absorption tail. More specifically,

$$F(R) = \frac{(1-R)^2}{2R}.$$

In the Urbach tail region, where the absorption coefficient α follows an exponential dependence on the photon energy, the Kubelka-Munk function can be used as an approximation for α. Taking the natural logarithm of $F(R)$ we obtain:

$$\ln(F(R)) \propto \frac{h\nu}{E_U},$$

where $h\nu$ is the photon energy. Thus, plotting $\ln((F(R))$ as a function of the photon energy in the spectral region just below the band edge for the three lead-free perovskites studied in this work yields a linear region with slope given by the inverse of the Urbach energy, see Figure S1. We find that E_U is 120 meV (CSI), 103 meV (CSB-RT) and 139 meV

(CSB-150).

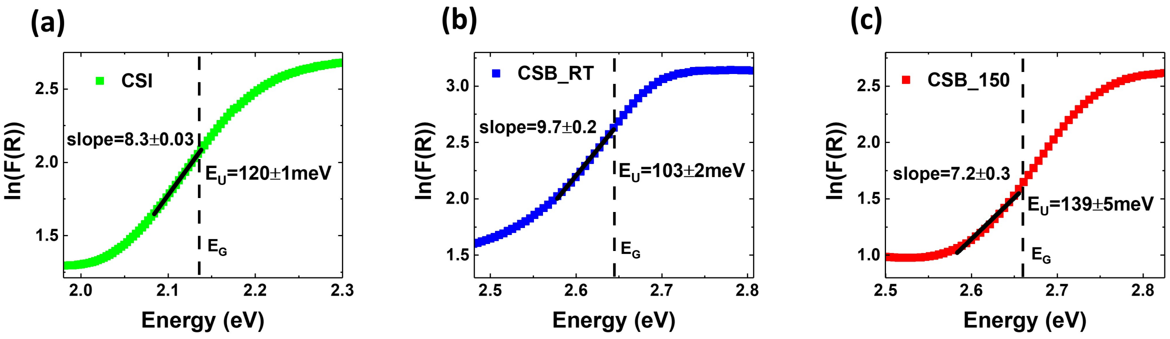

Figure 1: (a-c) Plots of $\ln(F(R))$ *vs.* energy for CSI, CSB-RT and CSB-150, respectively. The scattered points are measured data, whereas the continuous line is a fit from which the Urbach energy is extracted, see text.

S3. Irradiance dependence of the photocurrent and role of trap states

The device geometry studied in this work can be considered two-dimensional, since the current flows in a thin layer between two coplanar contacts defining a semiconducting channel of width two orders of magnitude larger than its length, and thickness more than one order of magnitude smaller than the channel length. In the presence of trap states near one of the band edges, in a photoconductor with non-injecting contacts, a large asymmetry of the electrons and holes mobilities leads to space-charge photocurrent at high irradiance.[8] Recent studies have shown that in 2D case the space charge limited photocurrent has a unique functional dependence on the photoexcited carrier generation rate (G), which is proportional to the irradiance, and the bias (V) given by $I_{ph} \propto (VG)^{\gamma_{2D}}$. The exponent γ_{2D} is a unique function of the Urbach energy $\gamma_{2D} = \frac{E_U/k_BT+1}{E_U/k_BT+1}$ and it exhibits a unique functional dependence for different dimensionality of the system.[9,10] Since the Urbach energy is independently determined from diffuse reflectance measurements, in the case of space charge limited photocurrent with trap states inducing asymmetric transport the irradiance

dependence of the photocurrent is expected to be fully governed by the exponent γ_{2D}. Upon applying this analysis to the perovskites studied in this work, we find values of γ_{2D} of 0.83 and 0.86 for CSB-RT and CSB-150, respectively. These are in good agreement with the values found from the irradiance dependence of the photocurrent, confirming the role of trap states on the space charge limited photocurrent in these devices even at low irradiance. At high irradiance, bimolecular recombination is dominant and γ is closer to 0.5.[8] However, CSI photodetectors present different transport mechanisms with shallow traps dominating throughout the range of studied irradiance. In this case, the measured S-shaped photocurrent *vs.* irradiance is due to the filling of shallow traps at moderate irradiance.[11]

S4. $Cs_3Sb_2Br_9$ and $Cs_3Sb_2I_9$ photodetectors

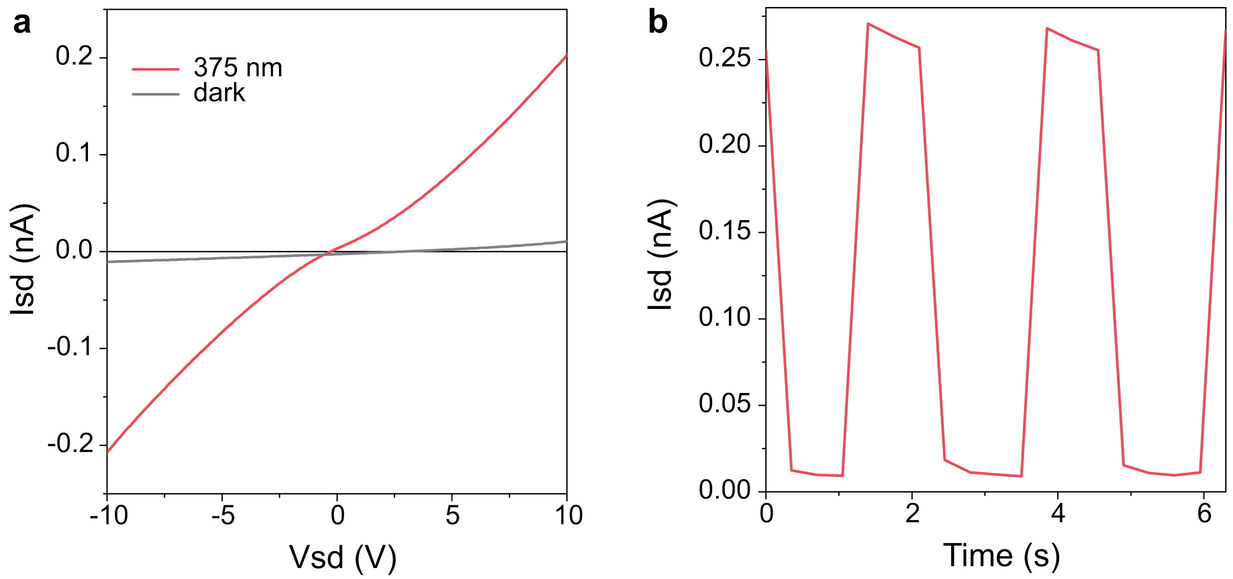

Figure 2: (a) Plots of current vs. voltage characteristics for CSB-RT photodetector measured in the dark (gray) and under continuous wave laser illumination (red) (b) Photoresponse of CSB-RT photodetector in (a) under pulsed laser illumination with a frequency of 1 Hz. ($\lambda = 375$ nm, irradiance I=1.09×10^{-4} W/cm^2, $d_\lambda \sim 490\,\mu$m and $A_{ph} = 2.2\times10^{-4}$ cm^2). The photoresponsivity of the same device exhibits slight variations depending on the illumination conditions, which can be attributed to reduced heating under modulated illumination. In the case of continuous irradiation R = 8.3 mA/W while by using pulsed irradiation R = 10 mA/W.

A similar increase in photocurrent upon air exposure has been reported in several perovskite systems and has been consistently attributed to oxygen-induced defect healing.[13–15] In Pb-based perovskites, oxygen molecules have been shown to bind strongly to halogen vacancies, which possess low formation energies and constitute the dominant intrinsic defect species. In the absence of passivation, these vacancies act as efficient charge-trapping and non-radiative recombination centres. Oxygen adsorption therefore suppresses trap-assisted recombination, resulting in improved optoelectronic performance, including longer carrier lifetimes and increased photocurrent.[13–15] Indeed, similarly to our observations, Mantulnikovs *et al.*[15] report a marked increase in photocurrent upon oxygen exposure, followed by a reduction after prolonged exposure attributed to the onset of material degradation of $MAPbbBr_3$ and $MAPbI_3$.

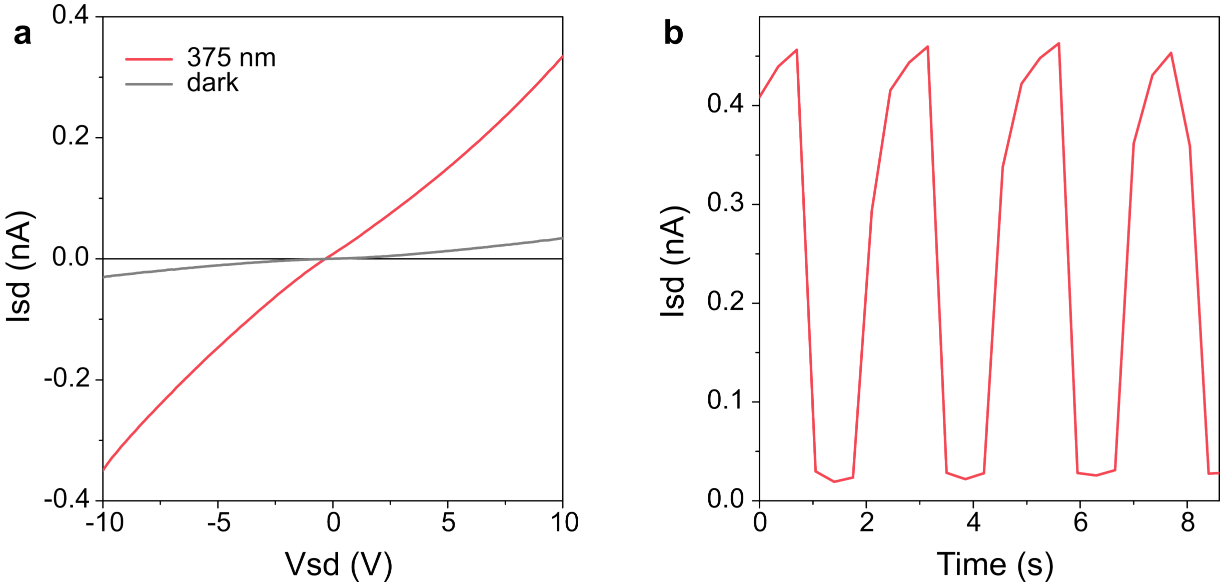

Figure 3: (a) Plots of current vs. voltage characteristics for CSB-150 photodetector measured in the dark (gray) and under continuous wave laser illumination (red) (b) Photoresponse of CSB-150 photodetector in (a) under pulsed laser illumination with a frequency of 1 Hz. ($\lambda = 375$ nm, irradiance I=1.09×10^{-4} W/cm^2, $d_\lambda \sim 490\,\mu$m and $A_{ph} = 2.2\times10^{-4}\,$cm^2). Photoresponsivity for the same device under continuous irradiation is R = 15.5 mA/W while under pulsed irradiation R = 18.3 mA/W.

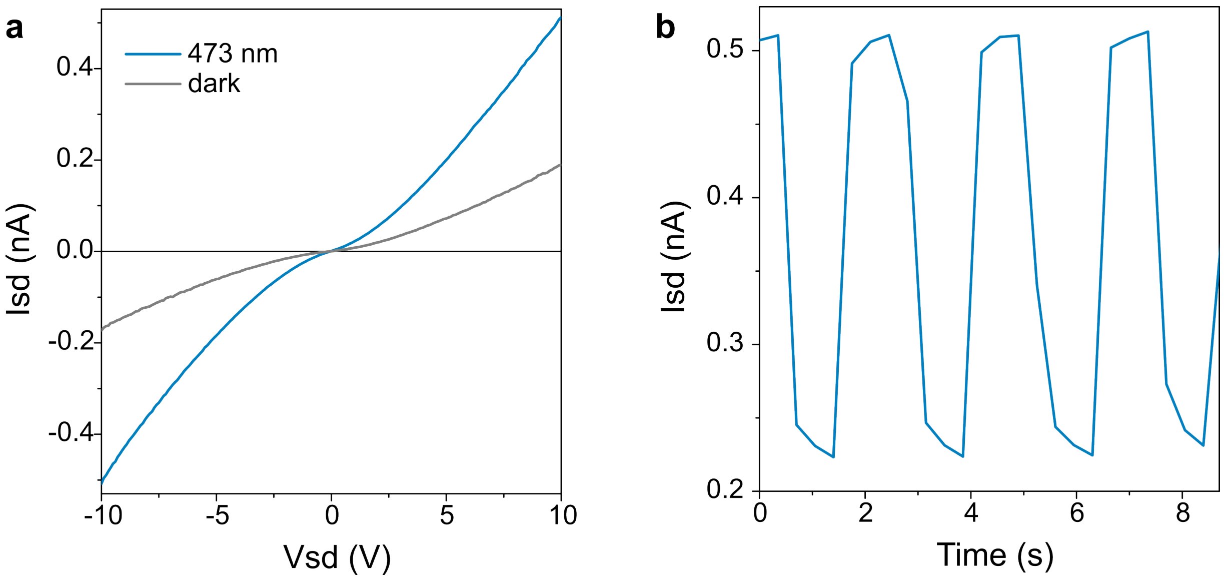

Figure 4: (a) Plots of current vs. voltage characteristics for CSI photodetector measured in the dark (gray) and under continuous wave laser illumination (light blue) (b) Photoresponse of CSI photodetector in (a) under pulsed laser illumination with a frequency of 1 Hz. ($\lambda = 473$ nm, irradiance I=2.64×10^{-6} W/cm^2, $d_\lambda \sim 600\,\mu$m and $A_{ph} = 2.9\times10^{-4}\,$cm^2). Photoresponsivity for the same device under continuous irradiation is R = 430 mA/W while under pulsed irradiation R = 470 mA/W.

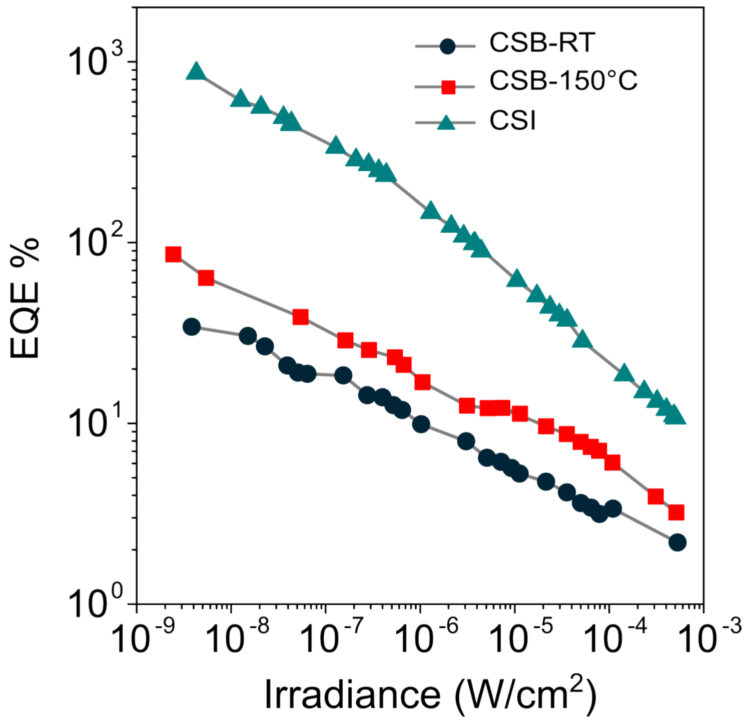

Figure 5: External quantum efficiency of CSB-RT, CSB-150, and CSI photodetectors.

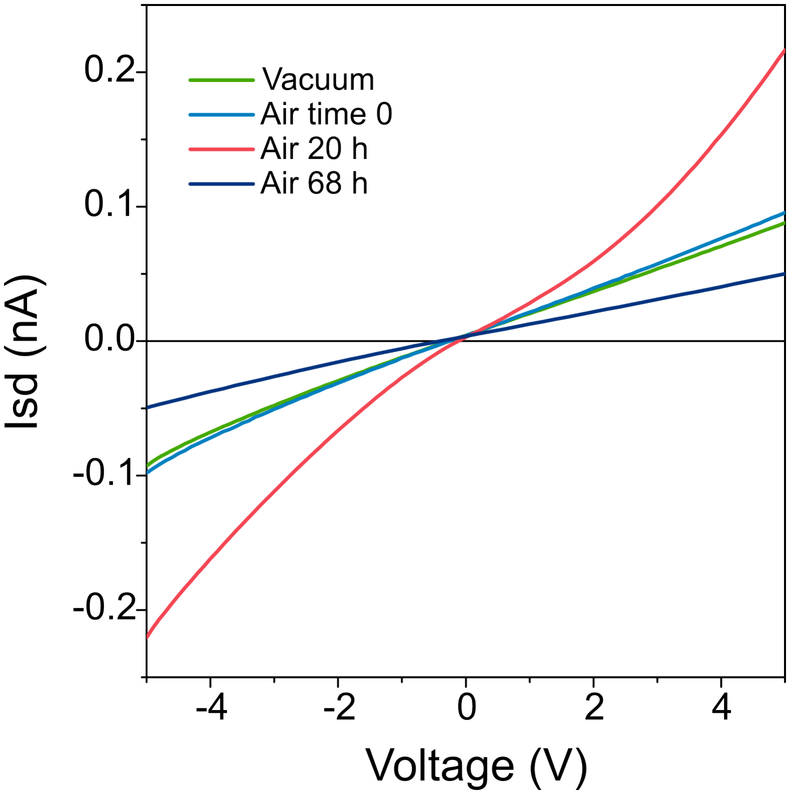

Figure 6: **Air stability of CSB-150 device**. Current vs. Voltage curves for CSB-150 photodetector measured in vacuum and ambient conditions, RH = 40% T = 23°C. ($\lambda = 375$ nm, irradiance $I = 1.09\times10^{-4}$ W/cm^2, $d_\lambda \sim 490\,\mu$m and $A_{ph} = 2.2\times10^{-4}$ cm^2). The device remains stable under ambient conditions and demonstrates a noticeable increase in photocurrent following exposure to air, likely due to oxygen passivation.[12] Even after 68 hours in air, the device continues to exhibit photoresponse, despite a slight decrease in performance that can be attributed to long-term degradation effects.

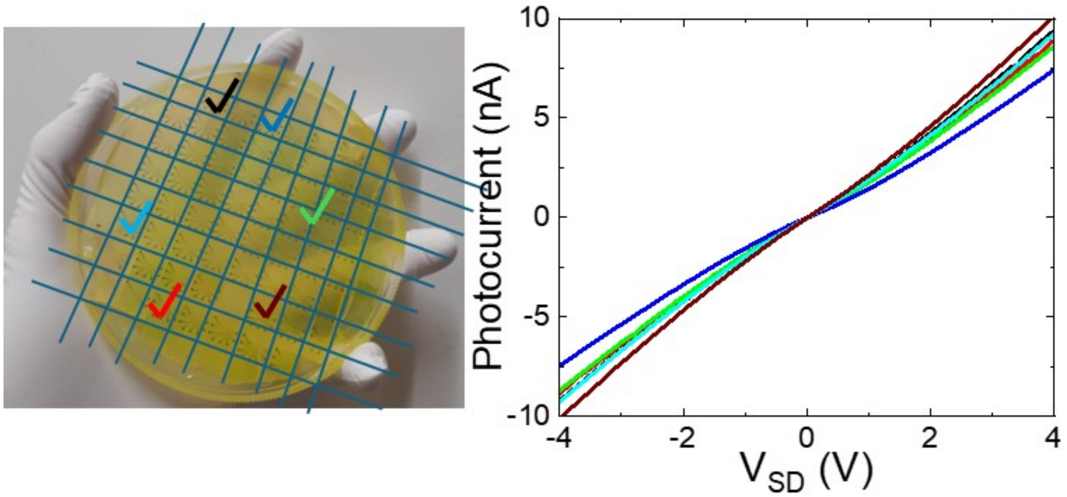

Figure 7: **Photocurrent spread**. Photocurrent measurements of devices from various different parts of the wafer as shown by the coloured ticks. A spread of less than 14% is observed when illuminating the devices with a white light LED at fixed irradiance of $10\,\mathrm{mW/cm^2}$.

Table 2: Performance comparison between lead-free Sb-based perovskite photodetectors based on single crystals (sc), polycrystalline (poly), and microplates (micro)

Material	Type	Detectivity (Jones)	Responsivity (mA/W)	Rise time (ms)	Fall time (ms)	LDR(dB)	Ref
$Cs_3Sb_2Br_9$	sc	3.77×10^{12}	2290	0.2	3.0	-	Liu 2020
$Cs_3Sb_2Br_9$	sc	2.60×10^{12}	3800	48	24	-	Zheng 2019
$Cs_3Sb_2I_9$	poly	4.30×10^{12}	62.0	0.106	0.085	51.3	Mei 2021
$Cs_3Sb_2I_{9-x}Cl_x$	poly	6.10×10^{12}	205	0.018	0.012	83.3	Mei 2021
$Rb_3Sb_2I_9$	poly	3.50×10^{12}	174	0.106	0.095	83.3	Mei 2021
$(MA)_3Sb_2I_9$	sc	1.00×10^{13}	40000	0.4	0.9	84	Yang 2018
$(MA)_3Sb_2Br_9$	sc	5.00×10^{8}	0.03	1000	1000	-	Yang 2018
$Cs_3Sb_2Br_9$	micro	1.00×10^{10}	36.9	61.5	24	-	Shil 2021
$Cs_3Sb_2I_9$	poly	4.30×10^{10}	54.5	50	30	-	Shil 2023
$Cs_3Sb_2Br_9$	poly	1.60×10^{10}	3.60	108	56.2	-	Shil 2023
$(MA)_3Sb_2Br_9$	sc	4.32×10^{11}	113	47.1	1162	67.96	Hun 2021
$(C_4H_{18}N)_6Sb_7I_{27}$	poly	2.00×10^{11}	15.0	0.600	3.00	102.3	Jia 2024
$Cs_3Sb_2I_9$	sc	6.80×10^{9}	12.2	46.0	10.0	-	Hong 2025
$Cs_3Sb_2I_9$	sc	1.10×10^{9}	178	36.0	21.0	-	Sheng 2025
$Cs_3Sb_2Br_9 - RT$	poly	1.23×10^{13}	110	0.034	0.045	163.9	This work
$Cs_3Sb_2Br_9 - 150°C$	poly	6.23×10^{13}	261	0.041	0.060	163.9	This work
$Cs_3Sb_2I_9$	poly	1.70×10^{15}	3290	0.032	0.038	165.4	This work

S5. Time response and bandwidth

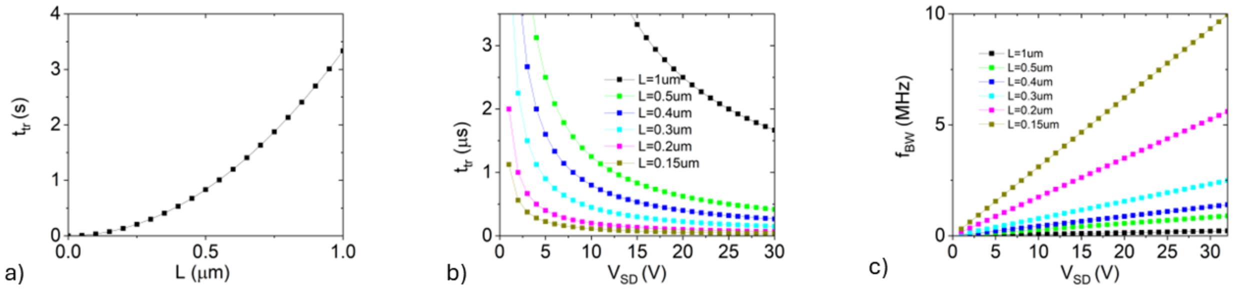

Figure 8: a) Predicted photodetector channel length dependence of the transit time for the devices measured in Fig. 4 of the main manuscript. b) Bias dependence for the transit time for different values of the channel length. c) Bias dependent bandwdidth for different values of channel length

In the studied photodetectors, the transit time t_{tr} of the photoexcited charge carriers in the perovskite channel is determined by the drift velocity and the channel length. Since the drift velocity is a function of the applied electric field and charge carrier mobility, we can express $t_{tr} = L^2/\mu V_{SD}$ as previously shown.[16,17] The time response measured in the L=3 μm perovskite photodetectors is t_{tr} ≈30 μs at $V_{SD} = +15V$. Hence, the channel length dependence of t_{tr} can be expressed as $t_{tr} = L^2 \times 30\mu s/(3\mu m)^2$, see Fig. 7b. On the other hand, the bias dependence of the transit time and the bandwidth ($f_{BW} = 0.35/t_{tr}$) of the photodetectors for different channel lengths are shown in Fig. 7b and c, respectively.

S6. Specific Detectivity Calculation

The specific detectivity (D^*) was calculated using the standard relation:

$$D^* = \frac{R \cdot \sqrt{A}}{I_{n,highest}} \tag{2}$$

where R is the measured responsivity (A/W), A is the active device area (cm^2), and $I_{n,highest}$ is the noise current density (A/Hz$^{1/2}$), which for consistency across devices and to avoid underestimation, we selected the highest measured noise within the frequency range measured,

carried out using the Rohde & Schwarz FSU Spectrum Analyzer.

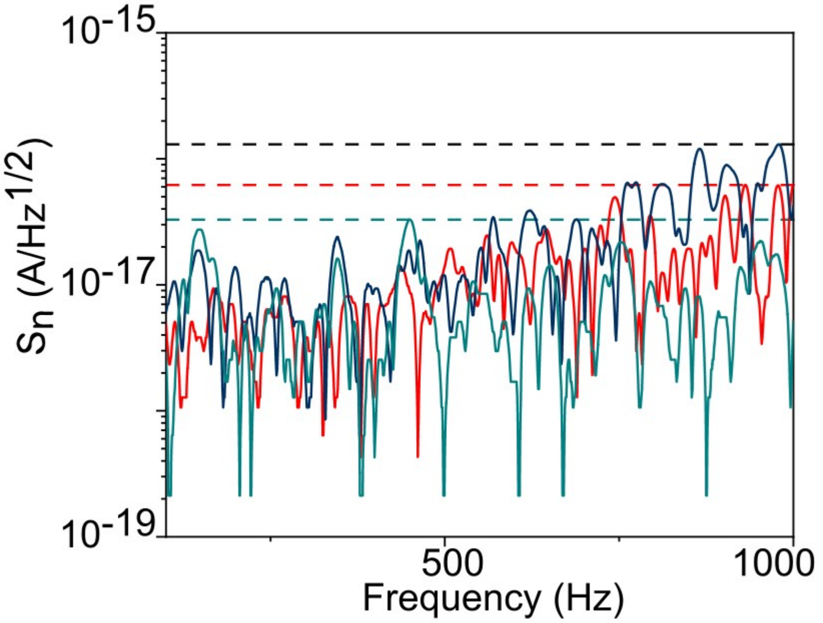

Figure 9: Plot of the measured noise spectral density for the devices in the main manuscript.

References

1. Kresse, G.; Furthmüller, J. Efficiency of Ab-Initio Total Energy Calculations for Metals and Semiconductors Using a Plane-Wave Basis Set. *Computational Materials Science* **1996**, *6*, 15–50.

2. Kresse, G.; Furthmüller, J. Efficient Iterative Schemes for *Ab Initio* Total-Energy Calculations Using a Plane-Wave Basis Set. *Physical Review B* **1996**, *54*, 11169–11186.

3. Perdew, J. P.; Burke, K.; Ernzerhof, M. Generalized Gradient Approximation Made Simple. *Physical Review Letters* **1996**, *77*, 3865–3868.

4. Krukau, A. V.; Vydrov, O. A.; Izmaylov, A. F.; Scuseria, G. E. Influence of the exchange screening parameter on the performance of screened hybrid functionals. *The Journal of Chemical Physics* **2006**, *125*, 224106.

5. Kim, S.; Lee, M.; Hong, C.; Yoon, Y.; An, H.; Lee, D.; Jeong, W.; Yoo, D.; Kang, Y.; Youn, Y.; Han, S. A band-gap database for semiconducting inorganic materials calculated with hybrid functional. *Scientific Data* **2020**, *7*, 387.

6. Monkhorst, H. J.; Pack, J. D. Special points for Brillouin-zone integrations. *Physical Review B* **1976**, *13*, 5188–5192.

7. Batatia, I.; Benner, P.; Chiang, Y.; Elena, A. M.; Kovács, D. P.; Riebesell, J.; Advincula, X. R.; Asta, M.; Baldwin, W. J.; Bernstein, N.; Bhowmik, A.; Blau, S. M.; Cărare, V.; Darby, J. P.; De, S.; Pia, F. D.; Deringer, V. L.; Elijošius, R.; El-Machachi, Z.; Fako, E. *et al.* A foundation model for atomistic materials chemistry. **2023**, arXiv:2401.00096.

8. Hahn, T.; Tscheuschner, S.; Kahle, F. J.; Reichenberger, M.; Athanasopoulos, S.; Saller, C.; Bazan, G. C.; Nguyen, T. Q.; Strohriegl, P.; Bässler, H.; Köhler, A.

Monomolecular and Bimolecular Recombination of Electron–Hole Pairs at the Interface of a Bilayer Organic Solar Cell. *Advanced Functional Materials* **2017**, *27*, 1604906.

9. De Visschere, P.; Woestenborghs, W.; Neyts, K. Space-charge limited surface currents between two semi-infinite planar electrodes embedded in a uniform dielectric medium. *Organic Electronics* **2015**, *16*, 212–220.

10. Hartnagel, P.; Kirchartz, T. Understanding the Light-Intensity Dependence of the Short-Circuit Current of Organic Solar Cells. *Advanced theory and simulations* **2020**, *3*, 2000116.

11. Zeiske, S.; Li, W.; Meredith, P.; Armin, A.; Sandberg, O. J. Light intensity dependence of the photocurrent in organic photovoltaic devices. *Cell Reports Physical Science* **2022**, *3*, 101096.

12. Liu, S.-C.; Li, Z.; Yang, Y.; Wang, X.; Chen, Y.-X.; Xue, D.-J.; Hu, J.-S. Investigation of Oxygen Passivation for High-Performance All-Inorganic Perovskite Solar Cells. *Journal of the American Chemical Society* **2019**, *141*, 18075–18082, PMID: 31638806.

13. Szemjonov, A.; Galkowski, K.; Anaya, M.; Andaji-Garmaroudi, Z.; Baikie, T. K.; Mackowski, S.; Baikie, I. D.; Stranks, S. D.; Islam, M. S. Impact of Oxygen on the Electronic Structure of Triple-Cation Halide Perovskites. *ACS Materials Letters* **2019**, *1*, 506–510.

14. Huang, L.; Ge, Z.; Zhang, X.; Zhu, Y. Oxygen-induced defect-healing and photo-brightening of halide perovskite semiconductors: science and application. *Journal of Materials Chemistry A* **2021**, *9*, 4379–4414.

15. Mantulnikovs, K.; Glushkova, A.; Kollár, M.; Forró, L.; Horváth, E.; Sienkiewicz, A. Differential Response of the Photoluminescence and Photocurrent of Polycrystalline CH3NH3PbI3 and CH3NH3PbBr3 to the Exposure to Oxygen and Nitrogen. *ACS Applied Electronic Materials* **2019**, *1*, 2007–2017.

16. Jones, G. F.; Pinto, R. M.; De Sanctis, A.; Nagareddy, V. K.; Wright, C. D.; Alves, H.; Craciun, M. F.; Russo, S. Highly Efficient Rubrene–Graphene Charge-Transfer Interfaces as Phototransistors in the Visible Regime. *Advanced Materials* **2017**, *29*, 1702993.

17. Mehew, J. D.; Unal, S.; Torres Alonso, E.; Jones, G. F.; Fadhil Ramadhan, S.; Craciun, M. F.; Russo, S. Fast and Highly Sensitive Ionic-Polymer-Gated WS2–Graphene Photodetectors. *Advanced Materials* **2017**, *29*, 1700222.
